# Developing and validating a Domain-specific Grit Scale for College Athletic Students

**DOI:** 10.1038/s41598-024-62771-z

**Published:** 2024-05-24

**Authors:** Feng Gao, Qiang Wei, Xiyue Dong, Jing Gao, Shan Lu, Yang Liu

**Affiliations:** 1 Qinggong College, North China University of Science and Technology, Qinhuangdao, China; 2 Department of Physical Education, Tangshan Normal University, Tangshan, China; 3 Department of Basic Courses, Tangshan Polytechnic College, Tangshan, China; 4 College of Physical Education, Yanshan University, Qinhuangdao, China

**Keywords:** Domain-specific Grit Scale, College athletic students, Scale development, Scale validation, Measurement invariance, Health policy, Risk factors

## Abstract

The aim of this study was to create and validate a ten-item Domain-specific Grit Scale for College Athletic Students (DGSCAS) to assess the level of grit among college athletic students. College athletic students from a single independent college located in a northern city in China (526 participants at time 1 and 589 participants at time 2) were assessed according to the scale. Various analyses were conducted in this study, including exploratory factor analysis (EFA), confirmatory factor analysis (CFA), and measurement invariance analysis across different sex and birthplaces. The results of the EFA revealed two factors: consistency of interests and perseverance of effort. The CFA results demonstrated acceptable fit indices (*x*^2^ = 160.048, *df* = 34, *x*^2^*/df* = 4.707, CFI = 0.983, TLI = 0.978, SRMR = 0.021, and RMSEA = 0.079). The scale exhibited satisfactory convergent validity and discriminant validity. The significant correlation of these factors with the Grit scale provided strong evidence of criterion-related validity. Measurement invariance analysis indicated that the scale performed consistently across different sex and birthplaces. Three limitations and corresponding recommendations were discussed, including sample heterogeneity, the lack of a unified test result as a criterion for predictive validity, and the cross-sectional design of the study. In conclusion, the DGSCAS is a practical and validated instrument that can be used to assess the level of grit among college athletic students in an educational context.

## Introduction

Grit^1^ has been conceptualized as an individual attribute that represents a person's passion and perseverance for achieving long-term goals. It reflects individuals' unwavering commitment and sustained effort when confronted with difficulties, as well as their ability to maintain focus and passion toward specific goals or interests. It is typically portrayed as a two-dimensional construct characterized by ‘perseverance of effort’ and ‘consistency of interests’. Perseverance of effort refers to the tendency to maintain commitment and sustain effort in times of difficulty. Consistency of interests refers to the ability to stay focused and passionate on a specific interest and goal over a long period of time. Together, perseverance of effort and consistency of interests capture the essence of grit, emphasizing both the tenacity to persevere through difficulties and the unwavering dedication and fascination toward one's goals or interests.

Extensive research has underscored the importance of grit across various domains, for instance, language learning^2–6^, mathematics^7,^^8–11^ and nursing^12–15^, and revealed its profound impact on individuals' resilience^16^, academic performance^17–24^, life satisfaction^25,26^, and well-being^27,28^. Recently, grit has garnered significant attention in the field of higher education, particularly within the context of university sports programs^29–33^.

As an emerging construct, it must be conceptualized and measured before any further investigations^34^. Personality researchers have frequently acknowledged the potential measurement benefits of adopting domain-specific personality inventories to enhance predictive validity in specific achievement settings^35–37^. Similarly, Bandura^38^ highlighted the fact that personality traits and motivational constructs are often too general to predict specific behaviors sufficiently. In line with this proposition, Wigfield^39^ stresses that the domain specificity of constructs marks an important research desideratum. Although grit was originally conceptualized as a domain-general (i.e., global) personality disposition^1,40^, some researchers (e.g.^34,41–45^ have highlighted the need to determine whether grit should be conceptualized and measured as a domain-specific construct.

In the domain of sports education, existing grit measurement scales primarily employ a domain-general approach. A recent scoping review of 90 grit studies in sports psychology^29^ noted that 58% of research in sports has used the Short Grit Scale, a domain-general scale developed by Duckworth and Quinn^40^. Moreover, when studies employ the Short Grit Scale to measure grit in athletes, researchers have invariably chosen the domain-general version of the instrument (e.g.^31,46–49^). A student athlete is a student who participates in sports alongside their studies, while an athlete is someone involved in sports or athletic activities without the specific association with being a student. Student athletes often face a demanding and rigorous schedule that includes both academic and athletic commitments. They need to juggle their coursework, assignments, and exams with their training, practices, and competitions. In such a demanding environment, grit becomes crucial as it enables them to persevere and maintain their focus and determination despite the challenges and setbacks they may encounter. Therefore, student athletes face a unique set of challenges and pressures compared to athletes. They must balance their academic responsibilities with their athletic commitments, which can be demanding and time-consuming. In addition, student athletes have dual identities as both students and athletes. Their experiences and interpretations of grit may differ from those of athletes who do not have the same academic obligations. Therefore, by developing a scale specifically for student athletes, researchers can capture the nuances and unique aspects of grit in this population.

To assess the concept of grit in student athletes, it is crucial to primarily consider specific learning activities for student athletes. Therefore, it is imperative to recognize the unique educational context of student athletes and tailor the measurement of grit accordingly. Assessing their perseverance and passion for physical activities, sports, and overall athletic performance should be integral to capturing their grit levels effectively. By acknowledging the importance of the physical component in their educational experiences, researchers and educators can gain a more comprehensive understanding of grit in this specific population. By aligning the measurement of grit with the physical and sports-related aspects of their education, we can better equip these students with the necessary skills and mindset to overcome obstacles and achieve long-term success in their athletic endeavors and overall academic journey.

Therefore, the primary objective of this study is to develop a domain-specific grit measurement scale for university sports programs. The significance of this research lies in advancing our understanding of grit in sports education. First, the development of a domain-specific grit measurement scale for university student athletes will not only contribute to the literature but also provide valuable insights for educators and practitioners in designing effective interventions and support systems for this specific population. This would enable targeted interventions and training programs to be developed, focusing on the specific dimensions of grit that are most relevant to athletic success. Second, adopting a domain-specific approach to measuring grit in sports education would also enhance the validity and reliability of the assessment. Ultimately, incorporating domain-specific measures of grit in sports education research can contribute to the advancement of theory and practice, supporting the development of resilient and determined athletes.

## Methods

### Data collection and participant description

The participants were collected using a convenience sampling method from a university located in northern China. This study was conducted with the approval of the university administration. The questionnaire was distributed electronically using Wenjuanxing, a popular survey platform in China. The current study utilized a two-wave data collection approach. Altogether, a total of 1115 college student majoring physical education were invited. The first wave was administered during the first week and involved a total of 526 participants. The demographic information of the participants was shown in Table 1. Specifically, in total, 417 participants were male and 109 were female. Of the participants, 121 came from urban areas, while 405 came from rural areas. With respect to academic status, 406 were first-year students, 118 were second-year students, and 2 were third-year students. The second wave took place in the fourth week and included 589 participants in total. Of the total number of participants, 440 were male and 149 were female. Of the participants, 138 were from urban areas, while 451 were from rural areas. Regarding academic status, 414 were first-year students, 90 were second-year students, and 85 were third-year students. The data from the first week were used to conduct exploratory factor analysis (EFA), while the data from the fourth week were used to conduct confirmatory factor analysis (CFA). The sample sizes for EFA and CFA were deemed adequate, as they met Nunnally's^50^ recommended ratio of at least ten respondents for every item. All students enrolled in this study are confirmed to be over 18 years old.

**Table 1:** Demographic information of the participants.

Demographic Variable	Category	EFA dataset		CFA dataset	
		Frequency	Percent	Frequency	Percent
Sex	Male	417	79.30	440	74.70
	Female	109	20.70	149	25.30
Valid	Urban	121	23.00	138	23.40
	Rural	405	77.00	451	76.60
Status	Junior	2	0.40	85	14.40
	Sophomore	118	22.40	90	15.30
	Freshman	406	77.20	414	70.30
Total		526	100.00	589	100.00

### Ethical considerations

In this study, ethical approval (Approval No.: OGXYLL20230011) was obtained from Qinggong College, North China University, to ensure the protection of human participants. The research protocol underwent a rigorous review process to ensure compliance with ethical standards and to safeguard the participants' rights and well-being. Prior to their involvement, informed consent was obtained from all participants. To facilitate this process, a digital consent form was integrated into the survey platform, Wenjuanxing. The consent form provided a clear explanation of the research purpose, procedures, potential risks and benefits and emphasized the voluntary nature of participation. Participants were assured of the confidentiality of their responses and the protection of their identities throughout the study. To ensure students' understanding and consent and the importance of informed consent, they were required to click the "Agree" button before accessing the questionnaire. Stringent measures were implemented to secure the collected data, restricting access so that it was only available to the research team. In order to prioritize the protection of student privacy, the collection of personally identifiable information, such as names and student IDs, was not mandated. Upholding the ethical principles of respect, beneficence, and justice, this study prioritized and protected the rights and well-being of college student athletes at all stages.

To conclude, the methods employed in this study were conducted in strict accordance with the ethical guidelines and regulations provided by Qinggong College, North China University of Science and Technology for research involving human participants. Informed consent was obtained from all participating university students via Wenjuanxing, and their confidentiality and privacy rights were protected throughout the study.

## Instruments

### Domain-specific Grit Scale for College Athletic Students (DGSCAS)

The development of the Domain-specific Grit Scale for College Athletic Students (DGSCAS) involved a multistep process beginning with operationalizing sports grit based on related literature and theories. This framework helps to identify the dimensions of sports grit that need to be measured in the questionnaire. In the current study, sports grit for college athletic students refers to the combination of passion for the sport (consistency of interests) and the ability to maintain a high level of effort and resilience in the face of challenges and setbacks (perseverance of effort) at the college level.

Next, preliminary questionnaire items were developed based on focus-group interviews with five students majoring in sports to measure each aspect of sports grit. Students were invited to answer questions with regard to the two proposed dimensions (consistency of interests and perseverance of effort). A total of ten frequently mentioned negative statements were used in the analysis of interview data. Grit is characterized by a person's ability to persevere and maintain long-term effort toward goals despite obstacles; therefore, using negatively worded items can specifically target and assess the challenges, setbacks, and difficulties individuals encounter in their pursuit of goals. In addition, employing negatively worded items allows for a more precise measurement of the negative aspects related to grit. It helps capture the extent to which individuals experience and overcome difficulties, setbacks, and failures in their pursuit of long-term goals. This specificity can provide a deeper understanding of the challenges individuals encounter and how they respond to them. The participants were directed to rate their degree of agreement with each statement on a five-point Likert scale, ranging from 1 (strongly disagree) to 5 (strongly agree). In the subsequent analysis, the data were reverse-coded accordingly.

Furthermore, a total of three sports teachers were invited to review the statements to provide feedback on their relevance, accuracy, and appropriateness for measuring the construct of interest. Based on the feedback, all items were deemed relevant statements that could be used as indicators of Sports Grit for College Athletic Students.

### Grit Scale

The Grit Scale is a 12-item scale developed by Duckworth, Peterson, Matthews, and Kelly^1^. The items were rated on a 5-point Likert scale (1 = Not like me at all and 5 = Very much like me). The statements were distributed in two factors, namely, Consistency of Interests and Perseverance of Effort. The "Consistency of Interests" was scaled according to six negatively phrased items (e.g., 'I often set a goal but later choose to pursue a different one'), and "Perseverance of Effort" was also scaled according to six items (e.g., 'I have achieved a goal that took years of work'). In the subsequent analysis, the data for the dimension "consistency of interests" were reverse-coded accordingly.

### Analytical procedure

First, an EFA was performed on the 10-item DGSCAS to establish its factorial structure^51^. The results from the EFA were subsequently utilized for CFA, which aimed to assess and improve the model's suitability.

Before conducting the EFA, Bartlett's test of sphericity and Kaiser‒Meyer‒Olkin (KMO) were employed to evaluate the normal distribution of the data and determine its suitability for factor analysis. Items were deemed appropriate for factor analysis if Bartlett's test yielded a statistically significant result and the KMO MSA value was equal to or greater than 0.80^52^. To determine the appropriate number of factors to extract, the study utilized both a scree plot and parallel analysis^53,54^. Parallel analysis is a method that compares the eigenvalues obtained from sample data with those expected from completely random data to determine the expected number of factors.

To statistically evaluate the factorial structure proposed by the EFA, a CFA was conducted on dataset B. Before conducting the CFA, both univariate and multivariate normality were assessed. Univariate normality was tested by examining the skewness and kurtosis of the data distributions. Skewness refers to the symmetry of the frequency distribution. Kurtosis is a statistical measure that describes the shape of a probability distribution or frequency distribution. According to Kline's^55^ recommendation, variables with absolute values of skewness greater than 3 and absolute values of kurtosis greater than 10 are considered problematic. Regarding multivariate normality, if Mardia's coefficient exceeded p(p + 2), where p represents the total number of observed indicators^56^, multivariate bootstrapping was employed.

To assess the construct validity, the study used the following criteria^57,58^: (1) the ratio of χ2 to degrees of freedom falls between 1 and 3,(2) the root mean square error of approximation (RMSEA) is less than 0.08; (3) the standardized root mean square residual (SRMR) is less than 0.05; (4) the Comparative Fit Index (CFI) is greater than 0.90; and (5) the Tucker‒Lewis Index (TLI) is greater than 0.95.

Convergent validity pertains to assessing the level of agreement among various measurements of a single concept and can be evaluated via different techniques, including standard factor loading, composite reliability (CR), and average variance extracted (AVE). A CR value exceeding 0.70 suggests dependable consistency across the measurements. To verify convergent validity, the AVE value should exceed 0.5, and the CR should be greater than the AVE, indicating that the observable variables accurately represent the underlying factor^59^.

The study assessed the discriminant validity using the Fornell-Larcker criterion method, which mandates that the square root of a construct's AVE must be higher than the correlation between the construct and any other constructs^60^. We also assessed the discriminant validity between the two dimensions of our scale using the Heterotrait-Monotrait ratio of correlations (HTMT). According to the recommendation by Henseler et al.^61^, the threshold for HTMT should be below 0.85 to ensure discriminant validity.

To obtain additional criterion-related validation evidence for the scale, we evaluated the correlations of the DGSCAS with participants’ responses to the grit scale^1^.

Two rounds of multigroup CFA were performed across participants of different sex (male and female) and birthplace (rural and urban) to examine whether the items on the test and the factorial structure of the research instrument were consistent across different groups. The aim was to ascertain whether the constructs remained consistent across the groups, thereby demonstrating that the construct validity could be applied in a general sense. We evaluated the scale's configural, metric, scalar, and residual invariance. The standard for determining invariance was a difference in the CFI or TLI of less than 0.01 and RMSEA less than 0.015, as recommended by Cheung and Rensvold^62^.

## Results

### Item level analyses

The findings of the item-level analyses are presented in Table 2. The results indicate that each item had a distribution that was roughly normal, as evidenced by the skewness and kurtosis values being less than ± 1. Furthermore, all items had a corrected item-total correlation greater than 0.3.

**Table 2:** Descriptive statistics for DGSCAS.

Item	Skewness	Kurtosis	M	SD	Corrected item-total correlation
T1F1	0.213	−0.563	3.048	1.049	0.709
T1F2	−0.135	−0.638	3.289	1.060	0.804
T1F3	−0.079	−0.624	3.308	1.058	0.832
T1F4	−0.048	−0.613	3.241	1.066	0.810
T1F5	−0.175	−0.674	3.435	1.079	0.784
T1F6	−0.289	−0.424	3.538	1.026	0.806
T1F7	0.090	−0.701	3.255	1.056	0.815
T1F8	0.012	−0.639	3.268	1.063	0.782
T1F9	−0.080	−0.584	3.327	1.056	0.788
T1F10	−0.095	−0.657	3.447	1.035	0.844

### Exploratory factor analysis

**Figure 1:**
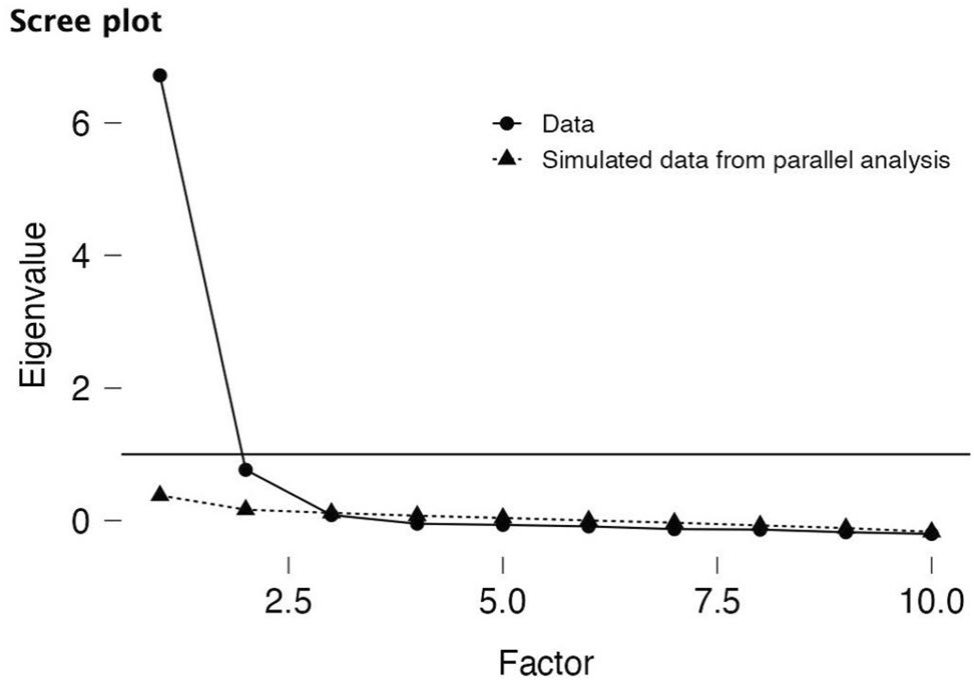
Scree plot for the two extracted factors.

JASP software^63^ was utilized to conduct EFA on the ten items. The KMO test, which is used to evaluate the suitability of the sample size, resulted in a value of 0.936, and Bartlett's test of sphericity resulted in a statistically significant value of χ^2^ = 5331.820 (p < 0.001), indicating that the sample was highly adequate for the analysis. Based on the results of parallel analysis and the scree plot shown in Fig. 1, two factors, *Consistency of interests* and *Perseverance of effort,* were identified, which accounted for a total of 77.0% of the variance. For the two-factor model, the absolute values of the loadings in Table 3 ranged from 0.731 to 0.912 for Factor 1, *Consistency of interests*, (accounting for a variance of 38.8%, α = 0.936), and from 0.673 to 0.934 for Factor 2, *Perseverance of effort*, (accounting for a variance of 38.2%, *α* = 0.947).

**Table 3:** Results of EFA of the 10‐item DGSCAS.

Factor	Name	Item	Factor 1	Factor 2	Uniqueness	Communality	Cronbach's alpha	Explained variance (%)
Factor 1	ConIn	T1F1	0.806		0.375	0.625	0.936	38.80
		T1F2	0.872		0.213	0.787		
		T1F3	0.912		0.150	0.850		
		T1F4	0.873		0.206	0.794		
		T1F5	0.731		0.305	0.695		
Factor 2	PerEff	T1F6		0.673	0.288	0.712	0.947	38.20
		T1F7		0.778	0.235	0.765		
		T1F8		0.934	0.197	0.803		
		T1F9		0.971	0.164	0.836		
		T1F10		0.821	0.170	0.830		

The applied rotation method is promax.

*ConIn* consistency of interests, *PerEff *perseverance of effort.

### Confirmatory factor analysis

#### Univariate and multivariate normality

For the test of univariate normality, since the absolute values of skewness were all lower than 3 and absolute values of kurtosis were lower than 10, as shown in Table 4, none of the variables were problematic^55^, therefore, the data could be treated as univariate normally distributed data. For the test for multivariate normality, the Mardia’s coefficient was 185.051, which was higher than the value of 120 computed based on the Formula p (p + 2), where p stands for the number of observed variables in the model^56^. As the data distribution did not conform to multivariate normality, we opted to report multivariate bootstrap instead of maximum likelihood (ML) inferences.

**Table 4:** Descriptive analysis.

Variable	Skew	C.r	Kurtosis	C.r
T2F1	0.199	1.97	−0.456	−2.258
T2F2	−0.064	−0.635	−0.482	−2.386
T2F3	−0.034	−0.338	−0.521	−2.579
T2F4	−0.03	−0.298	−0.446	−2.211
T2F5	−0.107	−1.058	−0.494	−2.446
T2F6	−0.212	−2.1	−0.392	−1.941
T2F7	0.022	0.222	−0.44	−2.18
T2F8	−0.058	−0.572	−0.531	−2.632
T2F9	−0.061	−0.608	−0.523	−2.59
T2F10	−0.179	−1.771	−0.412	−2.039
Multivariate			185.051	144.948

**Figure 2:**
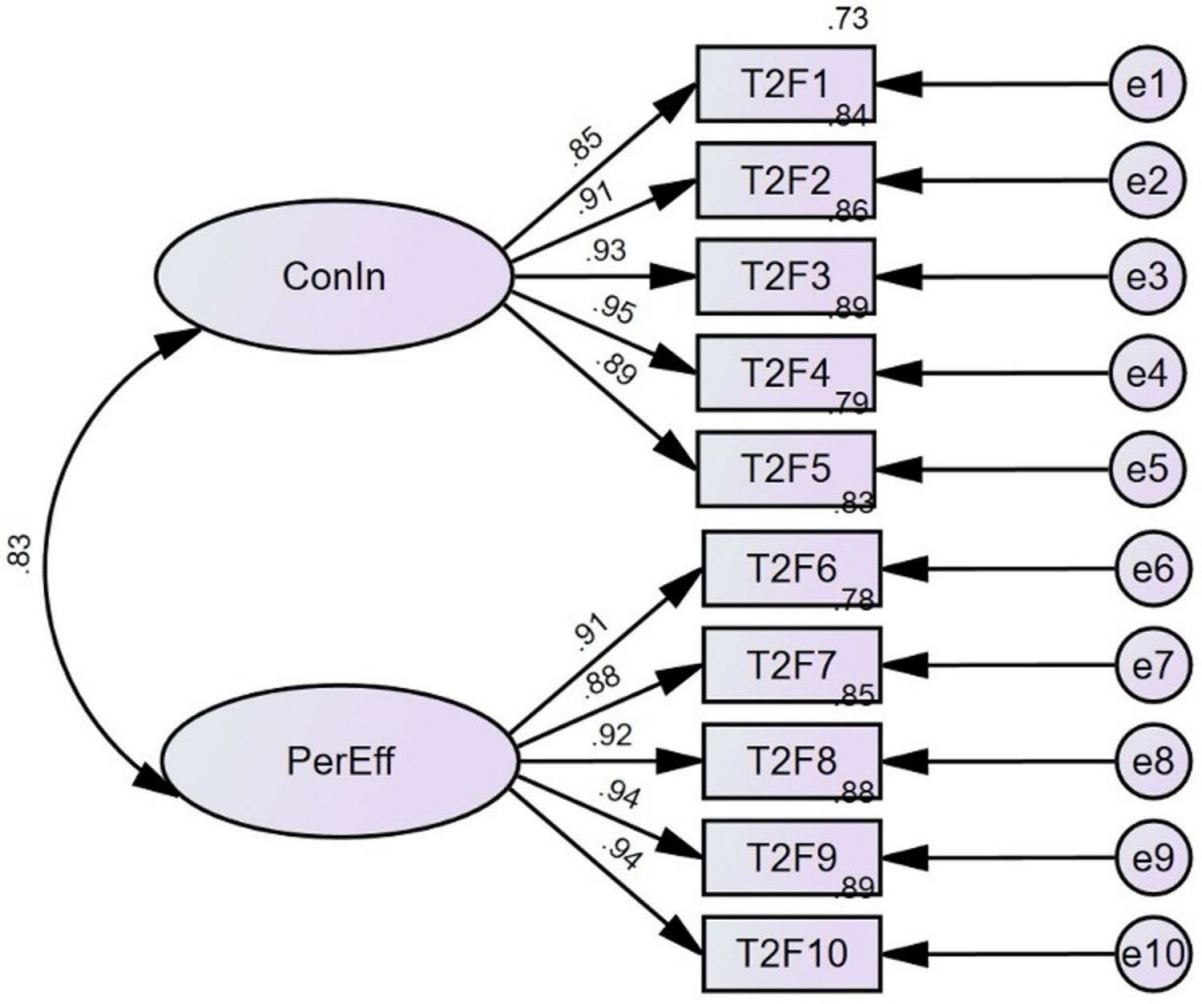
Results of the CFA of the two‐factor model of DGSCAS.

A confirmatory factor analysis (CFA) was conducted to validate the two-factor structure of the DGSCAS, as illustrated in Fig. 2. The results of the CFA, presented in Table 5, indicated that no item needed to be eliminated. The indices obtained, including × 2 = 160.048, df = 34, × 2/df = 4.707, CFI = 0.983, TLI = 0.978, SRMR = 0.021, and RMSEA = 0.079 (90% CI 0.067–0.092), suggested a good fit between the hypothesized two-factor model and the observed data.

**Table 5:** Fit indices for the DGSCAS.

Indices	χ^2^	df	χ^2^/df	RMSEA	SRMR	CFI	TLI
Acceptable fit	–	–	3–5	< 0.08	< 0.05	> 0.9	> 0.9
Two-factor model	160.048	34	4.707	0.079	0.021	0.983	0.978
One-factor model	1265.685	35	36.162	0.245	0.074	0.838	0.791

*DGSCAS* Sports Grit Scale for College Athletic Students, *RMSEA* root mean square error of approximation, *SRMR* standardized root mean squared residual, *CFI* comparative fit index, *TLI* Tucker–Lewis index.

Based on our research results, both exploratory factor analysis and confirmatory factor analysis support the two dimensions of grit. However, due to the relatively high covariance between these two dimensions (= 0.83), we conducted a model comparison. We included all the items in a general grit factor and compared the relevant indicators in the table below. After considering all the indicators, we ultimately selected the "two factor model" as the most suitable model for our study. This model can better explain the two dimensions of grit and also demonstrates good fit. According to the recommendation by Hair et al.^59^ the covariance between factors should be less than 0.85. Therefore, this further supports the notion that grit is a construct with two dimensions.

A high covariance suggests that college athletic students who display high levels of perseverance of effort also tend to exhibit high levels of consistency of interest. This can be attributed to the mutual reinforcement between these factors. Students who consistently show interest in a specific goal or activity in sports are more likely to persist in their efforts, while those who persevere despite challenges are more likely to sustain their interest in sports over time. Meanwhile, it is also important to consider contextual factors that can influence the covariance between these factors. For example, environmental elements such as coach support systems, opportunities for athletic growth, and the perceived value of the goal or activity can impact how perseverance and consistency are expressed in an individual's behavior and mindset.

### Convergent validity

After confirming the factor structure through CFA, we proceeded to assess the convergent validity of the DGSCAS to obtain additional evidence of its construct validity^64^. To assess convergent validity, the standard factor loading, composite reliability (CR), and average variance extracted (AVE) were calculated, with values greater than 0.50, 0.70, and 0.50 being considered acceptable, respectively^59^. As shown in Fig. 2, all items displayed sufficiently high loadings on their corresponding constructs, ranging from 0.85 to 0.95, all higher than the recommended cutoff value of 0.50. The CR values for the two factors (presented in Table 6) were 0.958 and 0.965, respectively, surpassing the threshold value of 0.70. In addition, the AVE for the two factors, which were 0.821 and 0.846, exceeded the cutoff point of 0.50 and were all smaller than the CR values. These findings suggest acceptable convergent validity of the DGSCAS.

**Table 6:** Convergent validity of confirmatory factor analysis.

	No. of items	CR	AVE	
ConIn	5	0.958	0.821	
PerEff	5	0.965	0.846	

*CR* composite reliability, *AVE* average variance extracted, *ConIn* consistency of interests, *PerEff* perseverance of effort.

### Discriminant validity

The Fornell–Larcker discriminant validity index provided support for the discriminant validity of the DGSCAS. Discriminant validity was established as the squared correlations between the constructs were found to be less than the AVE value for each construct, as proposed by Fornell and Larcker^60^. The summary of the discriminant validity index, presented in Table 7, showed that the bolded square root values of the AVE were greater than the off-diagonal correlation values between subconstructs. The AVE values were higher than the interconstruct correlations, indicating that the two factors were distinct.

**Table 7:** Fornell–Larcker discriminant validity of DGSCAS.

	ConIn	PerEff
ConIn	**0.906**	
PerEff	0.833***	**0.920**

*ConIn* consistency of interests, *PerEff* perseverance of effort.

***Correlation is significant at the 0.01 level (2-tailed).

Significant values are in bold.

In our study, we also assessed the discriminant validity between two dimensions using the Heterotrait–Monotrait ratio of correlations (HTMT). To determine whether discriminant validity is violated, we compared the HTMT value to the threshold of 0.85, as suggested by Henseler et al.^61^. Our analysis revealed that the HTMT value between the two dimensions was 0.834 as shown in Table 8, which is significantly below the threshold of 0.85. This indicates that the correlation between the two dimensions is sufficiently lower than the correlation within each dimension, supporting the discriminant validity of our measurement tool. It suggests that the two dimensions are distinct and can be reliably measured as separate constructs.

**Table 8:** HTMT analysis.

	ConIn	PerEff
ConIn		
PerEff	0.834	

### Criterion-related validity

In the current study, the criterion-related validity of the DGSCAS was evaluated by the Pearson correlation coefficients with the 12-item grit scale^1^. The results in Table 9 show that the two factors and the average score of the DGSCAS were significantly and positively correlated with the Grit Scale. These correlations provided robust evidence for the concurrent validity of the two-factor construct of the DGSCAS.

**Table 9:** Correlation coefficients between the DGSCAS and the 12-item Grit scale.

	ConIn	PerEff	DGSCAS	Grit
ConIn				
PerEff	.801**			
DGSCAS	.948**	.950**		
Grit	.448**	.526**	.514**	

*DGSCAS* Sports Grit Scale for College Athletic Students, *ConIn* consistency of interests, *PerEff* perseverance of effort, *Grit* 12-Item Grit Scale.

**Correlation is significant at the 0.01 level (2-tailed).

### Multigroup CFAs

Following the assessment of fit for the individual samples, two rounds of multigroup CFA were conducted to assess the measurement invariance of the DGSCAS across students of different sex and with different birthplaces. The outcomes presented in Tables 10 and 11 demonstrated that the measurement structure of the DGSCAS did not differ significantly across students of different sex and with different birthplaces, with a CFI and ΔTLI change of less than 0.010 and RMSEA less than 0.015.

**Table 10:** Measurement invariance between participants of different sex.

Model	Measurement Invariance and Model Comparison	χ2	*df*	χ2/df	Δχ2	Δ*df*	p	CFI	ΔCFI	TLI	ΔTLI	RMSEA	ΔRMSEA
M1 = unconstrained	–	305.870	68.000	4.498	–	–	–	0.969	–	0.959	–	0.077	–
M2 = measurement weights	Configural invariance = M2–M1	310.775	76.000	4.089	4.905	8.000	0.768	0.969	0.000	0.964	0.005	0.073	−0.004
M3 = measurement intercepts	Metric invariance = M3–M2	325.072	86.000	3.780	14.297	10.000	0.160	0.969	0.000	0.967	0.003	0.069	−0.004
M4 = Structural covariances	Scalar invariance = M4–M3	333.458	89.000	3.747	8.386	3.000	0.039	0.968	−0.001	0.968	0.001	0.068	−0.001
M5 = Measurement residuals	Residual invariance = M5–M4	384.284	99.000	3.882	50.826	10.000	0.000	0.963	−0.005	0.966	−0.002	0.070	0.002

**Table 11:** Measurement invariance among participants with different birthplaces.

Model	Measurement invariance and model comparison	χ2	*df*	χ2/df	Δχ2	Δ*df*	P	CFI	ΔCFI	TLI	ΔTLI	RMSEA	ΔRMSEA
M1 = unconstrained	–	236.818	68.000	3.483	–	–	–	0.978	–	0.971	–	0.065	–
M2 = measurement weights	Configural invariance = M2–M1	245.779	76.000	3.234	8.961	8.000	0.346	0.978	0.000	0.974	0.003	0.062	−0.003
M3 = measurement intercepts	Metric invariance = M3–M2	258.655	86.000	3.008	12.876	10.000	0.231	0.977	−0.001	0.976	0.002	0.058	−0.004
M4 = structural covariances	Scalar invariance = M4–M3	262.895	89.000	2.954	4.240	3.000	0.237	0.977	0.000	0.977	0.001	0.058	0.000
M5 = measurement residuals	Residual invariance = M5–M4	299.541	99.000	3.026	36.646	10.000	0.000	0.974	−0.003	0.976	−0.001	0.059	0.001

## Discussion

The purpose of this article was to describe the development of a new questionnaire to measure Chinese college student athletes’ grit, providing validity evidence based on the internal structure of the construct measured. The primary rationale for constructing a new instrument was the nature of the domain specificity of grit and the lack of a proper tool for college student athletes’ grit assessment. With regard to content validity, three highly professional sports teachers with extensive knowledge and expertise in conducting sports research were consulted, and the overall feedback was positive, and no further modification was suggested. The results of EFA revealed a two-component structure of the DGSCAS, which is similar to a two-component structure of the domain-general Grit–S.

The ten items loaded significantly on their corresponding subdimensions, providing evidence that the DGSCAS is multidimensional and is composed of two different subscales. These findings, therefore, confirm that the DGSCAS has good internal construct validity among Chinese student athletes, and as such, it is a reliable and valid instrument for measuring Chinese college student athletes’ grit.

Given that the newly designed instrument was developed to measure sports-specific grit, the EFA results can be interpreted as yielding robust evidence in favor of the construct validity of the DGSCAS. Rigorous validation procedures of CFA with a different sample corroborated the structure of grit for college student athletes, including consistency of interests and perseverance of effort with robust reliability and validity. In addition, significant correlations of the DGSCAS with the grit scale offered evidence of the criterion validity of the scale.

The findings indicate that the new instrument demonstrated a high degree of concurrent validity. Finally, measurement invariance by sex and birthplace was also tested. Strong invariance assures that the latent means can be compared by sex and birthplace. These analyses further support the factor structure of the DGSCAS and its utility for comparative research. In fact, factor loading invariance (i.e., weak invariance) allows for comparisons of the correlates^65^, while Steenkamp and Baumgartner^66^ suggest that two indicators with invariant loadings and intercepts (i.e., partial strong invariance) are adequate for mean comparisons.

The findings of this study revealed that the DGSCAS has adequate psychometric quality in terms of measurement reliability and validity. The CFA results provided empirical support for the existence of two separate yet related dimensions for the DGSCAS.

As a general conclusion, the analyses confirmed that the DGSCAS is a valid and reliable instrument for measuring Chinese college student athletes’ grit.

### Limitations and suggestions for future studies

This paper aims to develop and validate grit among Chinese college student athletes. However, it is important to acknowledge the limitations of the study to ensure a comprehensive understanding of the findings and provide valuable insights for future research.

One notable limitation of this study is the lack of sample heterogeneity. The participants were solely recruited from one college in Hebei Province, which may limit the generalizability of the findings to a broader population of Chinese college student-athletes. To address this limitation, future studies should aim to include participants from multiple colleges and regions across China. By incorporating a more diverse sample, researchers can obtain a broader representation of the Chinese college student-athlete population. Different regions may have unique educational systems, societal expectations, and sports infrastructure, which can impact the development and validation of grit among student-athletes. Future studies can enhance the external validity and generalizability of their findings. This will contribute to a better understanding of grit development among Chinese college student-athletes and provide valuable insights for educational institutions, sports organizations, and practitioners involved in the development and support of student-athletes.

Another limitation is the absence of a unified test result serving as a criterion for predictive validity. The inclusion of participants from different grades (freshmen, sophomores, and juniors) prevents the establishment of a common criterion to assess the predictive validity of the grit measurement. Future studies should consider implementing a longitudinal design to overcome this limitation. By following a cohort of college student athletes over time, researchers can examine how grit levels relate to future outcomes such as academic performance or athletic success. Collaboration with educational institutions and sports organizations can provide objective performance measures for evaluating the predictive validity of the grit measurement.

Finally, despite validating the measurement invariance across sex and birthplace and achieving satisfactory reliability during the development of the questionnaire, an important limitation of the study is the adoption of a cross-sectional design. Although the data were collected in two waves, identifiable information such as student IDs was not collected, preventing the assessment of test–retest reliability and longitudinal measurement invariance. To address this limitation, future studies should consider incorporating identifiable information, such as student IDs or other unique identifiers, during data collection. This would enable researchers to track individual participants over time and assess the stability of the questionnaire's measurements through test–retest reliability analysis. Longitudinal measurement invariance could also be examined by collecting data at multiple time points and assessing the equivalence of the measurement model across these time points.

## Conclusion

In conclusion, while this study contributes to the understanding of grit development among Chinese college student athletes, it has certain limitations that should be considered. Sample homogeneity limits the generalizability of the findings, while the lack of predictive validity and the adoption of a cross-sectional design restrict the ability to assess the long-term impact of grit and establish causal relationships. Future studies should address these limitations by incorporating a more representative sample, implementing a longitudinal design, and considering mixed-methods approaches. By addressing these limitations, researchers can enhance the validity and generalizability of findings, providing valuable insights into the development and validation of grit among Chinese college student athletes (Supplementary Information).

### Supplementary Information

Supplementary Information 1.

Supplementary Information 2.

## Data Availability

Data is provided within the supplementary information files.

## Appendix A: Domain-specific Grit Scale for College Athletic Students

**Table:**

Dimension	Item	Statement
ConIn	1	I often struggle to stay motivated to train for my sport, and frequently find myself skipping workouts or cutting them short
	2	When I do train for my sport, I often feel like I am just going through the motions and am not fully engaged in the process
	3	I tend to make excuses for why I can not train, even when I know it would be beneficial for my performance and overall success
	4	I have difficulty sticking to a consistent training schedule, and often fall behind in my training plan
	5	I find sports exercise to be a chore and not enjoyable, and would much rather spend my time doing other activities that I find more interesting or engaging
PerEff	6	When I face challenges or setbacks in my training, I tend to give up or lose motivation to continue
	7	I find it difficult to push myself past my comfort zone when I am training, and tend to stick with what I am already good at
	8	I am easily discouraged by negative feedback or criticism, and sometimes let it affect my motivation to continue training
	9	When I am not seeing immediate progress in my training, I tend to become frustrated and lose motivation to continue
	10	I often struggle to maintain focus and mental toughness during challenging training sessions, and sometimes give up before completing them

*ConIn* consistency of interests, *PerEff* perseverance of effort.
